# Design of an Implantable Device for Ocular Drug Delivery

**DOI:** 10.1155/2012/527516

**Published:** 2012-07-18

**Authors:** Jae-Hwan Lee, Ramana M. Pidaparti, Gary M. Atkinson, Ramana S. Moorthy

**Affiliations:** ^1^Department of Mechanical and Nuclear Engineering, Virginia Commonwealth University, Richmond, VA 23284, USA; ^2^Department of Electrical and Computer Engineering, Virginia Commonwealth University, Richmond, VA 23284, USA; ^3^Associated Vitreoretinal and Uveitis Consultants & Indiana University Medical Center, Indianapolis, IN 46202, USA

## Abstract

Ocular diseases, such as, glaucoma, age-related macular degeneration (AMD), diabetic retinopathy, and retinitis pigmentosa require drug management in order to prevent blindness and affecting million of adults in USA and worldwide. There is an increasing need to develop devices for drug delivery to address ocular diseases. This study focuses on the design, simulation, and development of an implantable ocular drug delivery device consisting of micro-/nanochannels embedded between top and bottom covers with a drug reservoir made from polydimethylsiloxane (PDMS) which is silicon-based organic and biodegradable polymer. Several simulations were carried out with six different micro-channel configurations in order to see the feasibility for ocular drug delivery applications. Based on the results obtained, channel design of osmotic I and osmotic II satisfied the diffusion rates required for ocular drug delivery. Finally, a prototype illustrating the three components of the drug delivery design is presented. In the future, the device will be tested for its functionality and diffusion characteristics.

## 1. Introduction

New drugs for treating eye diseases have been developed over the past decade and are very unique for each eye diseases, such as, glaucoma, cataracts, and age-related macular degeneration (AMD). It is estimated that 1.6 million adults in the USA over the age of 50 and above suffer from age-related macular degeneration and about 200,000 cases are diagnosed annually. Worldwide, about 500,000 cases are diagnosed annually [1]. Drugs currently utilized for AMD are delivered via repeated intravitreal injections of the drug into the eye. Risks of repeated intravitreal injections can include intraocular infections (endophthalmitis), intraocular hemorrhage, and retinal detachment. Also, reducing the frequency of dosing will clearly benefit the patient by reducing the need for risky intravitreal injections and improving the pharmacokinetics of the drug in the eye. Eye disease in the posterior segment includes two different forms of AMD, such as, dry and wet. Approximately 90% of patients with AMD have the Dry form shown in small yellow and white deposits form made of proteins and waste products. Wet ADM is caused by abnormal blood vessels grow out of the retina followed by rapid vision loss. However, these AMD diseases limit drug delivery in the retina region to eye drops [2, 3].

 The drug using a needle with syringe can be injected, but it barely provides the right amount of dose and over doses may cause more severe problems, such as, swelling, fatigue, and damage photoreceptor molecules. Furthermore, most drugs run out in a month and repeated injections become necessary. Developing an implantable drug delivery device will help reduce the costs and risks associated with frequent injections and facilitate delivering the drug in a controlled manner and in the required amounts and improve therapeutic efficacy and safety of drugs.

Ocular diseases, such as, glaucoma, age-related macular degeneration (AMD), diabetic retinopathy, and retinitis pigmentosa require drug management in order to prevent blindness [4]. These incurable diseases require lifelong treatment through orally administered medications, intraocular injections, and biodegradable implants. Drug delivery to ocular tissue is very difficult due to area and size limitations in the eye. There are currently at least three major categories of ocular drug delivery systems as discussed in [1]: biodegradable or nonbiodegradable, atypical implantable pump systems, and implantable pump systems. Implantable pump systems dispense drugs from an internal reservoir and have the advantage of providing control over drug delivery rate and volume. Several types of implantable pumps, such as, infusion pumps, osmotic pumps, and peristaltic pumps have been developed and used successfully for applications, such as, insulin delivery but have been found to be unsuitable for ocular drug delivery due to space limitations. Typical implantable pump systems (hydrogel systems infused with drug swell via intake of biological fluids for example) minimize the drug volume required for treatment and provide targeted delivery at a constant rate. However surgical procedures are required to implant and replace these devices, which may in turn contribute to additional side effects. Vitrasert and Retisert distributed by Bausch and Lomb are commercially available examples of nonbiodegradable systems. In these systems, the drug is released as a polymer matrix infused with drug dissolves or the drug is distributed from a nonbiodegradable reservoir. The disadvantages with these systems include in vivo polymer degradation (and the drug cannot be refilled) and the fact that drug delivery is dependent upon a limited volume available within the polymer.

Several models have been used to study the drug delivery mechanisms [5–7]. Recently, a review of barriers to posterior eye drug delivery and the challenges and opportunities were discussed by Thrimawithana et al. [8].  Table 1 summarizes various drugs, their diffusion coefficients, the average dosage, and the frequency to treat AMD diseases. Fick's second law of diffusion can be used to describe the transport of drug into the eye using microchannels. As the drug delivery device is implanted in the vitreous body of the eye, usually the diffusion depends on the local concentration rate between a drug reservoir and an aqueous humor. The Fick's second law of diffusion model can predict the diffusion time of the local tissue concentration in the eye following a variety of microchannel geometries for ocular implantable delivery. The diffusion coefficient of drugs may vary based on the chemical properties and internal structure as well as the molecular weight of the drug.

Several Micro Electro-Mechanical System (MEMS) devices, such as, microreservoirs and micropumps have been fabricated to address the spatial constraints posed by ocular drug delivery [1, 4]. Microreservoirs offer maximum control of drug delivery but cannot be refilled or reused, thereby ruling out suitability for treating chronic eye conditions. Peristaltic micropumps provide targeted drug delivery through active pumping but require considerable space to achieve a desired volume of flow per minute. To overcome these limitations, Lo et al. [9] recently developed a first generation prototype polymer MEMS delivery device with a refillable drug reservoir for treating ocular diseases. In addition to the refillable drug reservoir, the device consists of a transcleral cannula, check valve, and suture tabs. The device requires surgical implantation underneath the conjunctiva and the specified dose of medication is dispensed from the device when the reservoir is mechanically activated by the patient's finger. This device has several advantages when compared to existing systems including the following: the device is refillable, requires only a single surgical intervention, and is suitable for treating chronic ocular conditions; it is compact and fits within the dimensions imposed by the ocular orbit (<2 mm thick). However, the device requires patient's intervention in dispersion of the drug.

In order to cater to multiple scenarios in terms of amount of drug delivery and constraints, alternate MEMS devices might be of interest for treating ocular diseases. Nano-/microchannel-based drug delivery technologies represent an unprecedented opportunity to realize radically new devices that would exploit the novel features of the nanochannels, in which chip contains that drug reservoir with dose, provide unique performance in terms of diffusion and kinetics over existing technologies for drug delivery applications [10].

This study focuses on the design, simulation, and development of an implantable ocular drug delivery device. A novel design concept consisting of micro/nanochannels embedded between top and bottom covers with a drug reservoir made from PDMS material was developed. Several simulations were carried out with different microchannel configurations in order to see the feasibility for ocular drug delivery applications. Finally, a prototype illustrating the three components of the drug delivery design is presented.

## 2. Design and Development

### 2.1. Device Concept

**Figure 1:**
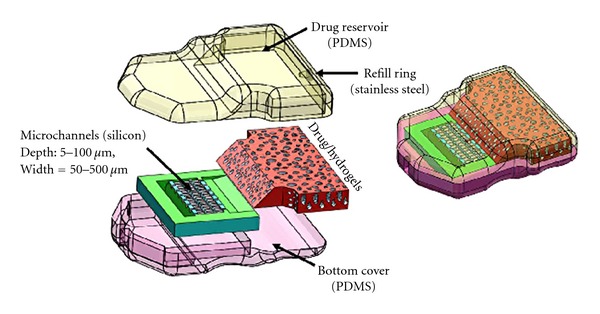
Proposed device design concept for ocular drug delivery.

**Figure 2:**
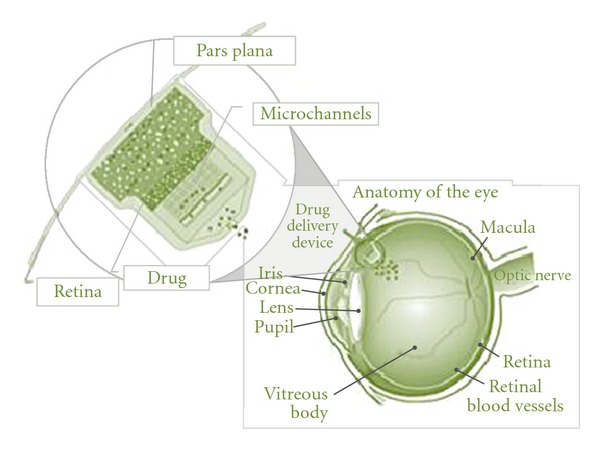
An overview of the attachment of the implanted drug delivery device to the eye.

A novel implantable device incorporating nano-/microchannels is proposed for ocular drug delivery. As shown in Figure 1, the drug is stored in a reservoir at one end of the device. Microchannels are coated with hydrophilic coatings so that the drug from the reservoir diffuses through the channels at specified/designed rate into the eye eliminating the need for any controlled actuation. Hydrogels (MIRAgel, MIRA Inc, Waltham, Mass), consisting of poly (methyl acrylate-Co-2-Hydroxyethyl acrylyte) are used as means to passively induce the drug delivery into the microchannels so that the drug diffuses freely through the channels and reaches the outlet for delivery. The microchannel component with inlet/outlet reservoirs will be enclosed in a PDMS case whose base is rounded to match the curvature of the eye globe. The device is attached securely to the sclera of the eye with fine 10–0 or 9–0 nylon sutures. Ideally, the device would be surgically, transclerally implanted in the vitreous space with an external thin curved spherical surface flange that would be nearly flush with the sclera and sutured in place (see Figure 2). The design requirements for the proposed drug delivery device are as follows:

-  target overall volume is less than 280 mm^3^;
-   diffusion rate is less than 0.07 nL/min;
-  target diffusion time period will be around 1 to 2 years;
-  kinetics: reliable diffusion coefficient of drugs through the microchannels;
-  implantable: eliminate repeated injections for effective treatment;
-  actuation: sustained release drug delivery methods.

### 2.2. Design Calculations

To illustrate the targeted volume and rate of the drug delivery device, the following section provides the details of calculations. It has been assumed that drug-contained deionized water will be transported through the microchannel from a reservoir. The corticosteroid fluocinolone acetonide has low solubility, so that solution was made by dissolving 59 mg of C_24_H_30_F_2_O_6_ in deionized water of 50 *μ*L (concentration in the device *≈* 1.18 mg/*μ*L). We also assume that the concentration of drugs in the water within the reservoir is around 1.18 mg/cm^3^ and zero concentration within the retina region of the eye. Using this value of concentration, we can estimate the flux density of drug-contained water transport into the retina region by molecular diffusion. However, in this study, we assume that the diffusion coefficient for typical eye drug, which is the corticosteroid fluocinolone acetonide in the deionized water, is equal to 2.3 × 10^−7^ cm^2^/s. The concentration of drug in the reservoir is very large in comparison to the concentration in the retina region. To calculate the flux density, we use Fick's Law (1), assuming that the gradient of concentration with length is linear over the microchannels path. The diffusive flux will be from the reservoir to the eye, from a high concentration to a lower concentration.

Fick's first law, which relates the diffusive flux to the concentration and is given as,

(1) $$\begin{array}{c} J=-D\;\frac{\partial \phi }{\partial x}, \end{array}$$

where, *J* is the diffusion flux (g/cm^2^ *·*s), *D* is the diffusion coefficient or diffusivity in dimension of cm^2^/s, and *ϕ* is the concentration of drugs in the reservoir.

Using, the above values, we get

(2) J=−  2.3  ×  10−7 cm2/s·(1.18  g/cm3/0.8 cm)=−  3.39  ×  10−7 g/(cm2)·s.

Ignoring the diffusion direction, we calculate the flux density of 3.39 × 10^−7^ g/cm^2^ · s and it can be used to calculate the total mass flux of drug into the eye using (3) given below. For example, if the straight microchannel has an inlet area of 0.0005 cm^2^ with 12 separate pathways, then the total flux into the eye is

(3) $$\begin{array}{c} M_{\text{total}}=J\times A, \end{array}$$

where, *A* is a section area at the inlet. 

Using the above values, we get

(4) Mtotal=3.39×10−7 g/cm2·s×0.0005 cm2×60 s/minute=1.02×10−8 g/min⁡  ≈1.04×10−4 μL/min⁡ or  2.58 mg/month         (total 12 microchannels).

As per our specification, the drug delivery device contains drug of 6 mg in the deionized water, it can be continuously used for around 11 to 12 months without refilling injection.

### 2.3. Analysis and Simulation

**Figure 3:**
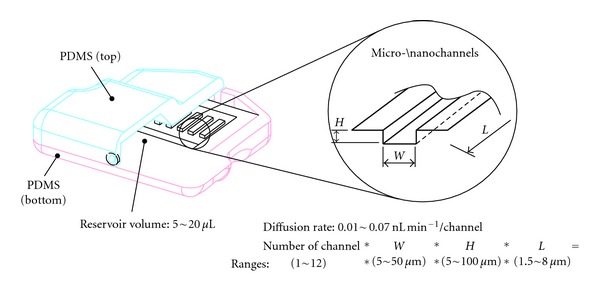
Schematic illustration of present device.

**Figure 4:**
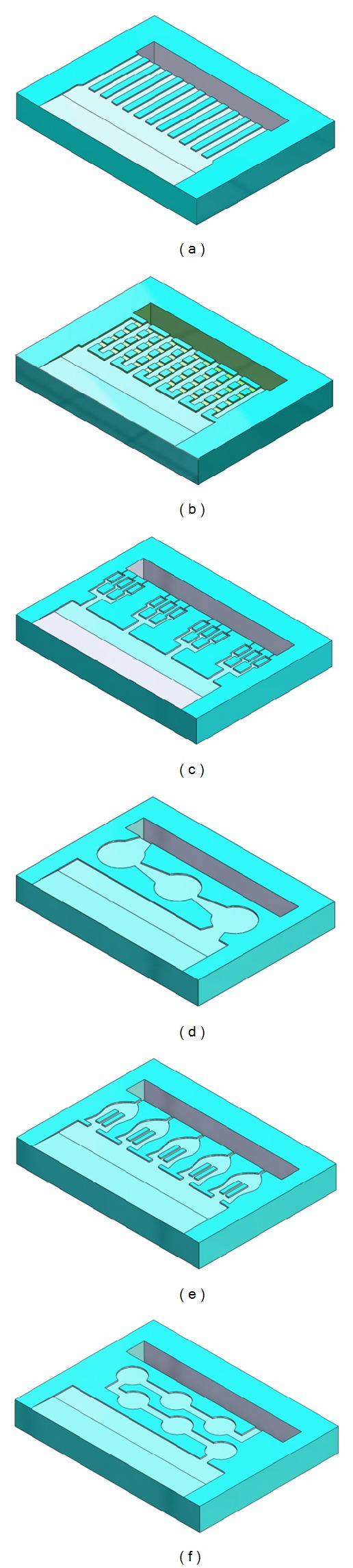
Various microchannels patterns considered for design analysis and simulation.

In order to illustrate the proof-of-concept, six different micro-/nanochannels are etched on the silicon substrate using photolithography technology. The overall dimensions of microchannels were within a range of 1.5 ~ 8.0 mm in length, had a depth of 5 to 100 *μ*m and a width may vary based on the geometry of microchannels (50 ~ 500 *μ*m) as shown in Figure 3. The length of microchannels depends on the geometry of diffusion channels. After the surface modification, such as, oxygen plasma, the channels will provide various diffusion rates in conjunction with the drug's diffusion coefficient. The injection cannula (needle gauge # 25 or 32) on the outlet of the device routes the drugs into the targeted region. In order to understand the design characteristics of the microchannels, we developed a coarse-grained representation of the microchannel geometry through computational fluid dynamic analysis and optimization. Specifically, the role of the microchannel geometry in passive free diffusion that molecules can pass freely through the microchannel follow concentration gradients is investigated and discussed. Finite element (FE) analysis using ANSYS-Multiphysics module was used to perform the design simulations. Six different microchannel geometries are developed to simulate the thermal diffusivity as shown in Figure 4. Drug concentration at the reservoir is assumed to be 1 kmol/m^3^ and drug concentration at the outlet to be 0 kmol/m^3^. This concentration represents drug concentration in the eye. Drug diffusivity is assumed to be the same as the synthetic corticosteroid fluocinolone acetonide in deionized (DI) water (2.3 × 10^−7^ cm^2^/s) [8].

Simulation results of drug diffusion within the microchannels as a function of time and drug diffusion from the drug reservoir revealed high-concentration areas to zero drug concentration areas in the vitreous body. Since drug consumption occurred at the blood vessel of a vitreous body which can be approximated as zero, the amount of diffusion may vary with the concentration gradient, however, a constant drug release rate can be obtained.

### 2.4. Fabrication

Master molds for both upper and bottom layers of the reservoir are made of Acura 50 plastic (3D system corp.) and constructed from 3D stereolithography process using 3D Viper SLA system (3D system corp.). PDMS [11] is mixed silicone elastomeric base and a curing agent with a 10 : 1 ratio (SYLGARD 184, DOW CORNING) and poured into the master molds. The PDMS is degassed in a vacuum machine for 20 minutes (Durable medical equipment Inc., Richmond, VA) and cured at room temperature for 24 hours or 80°C for 2 hours. The microchannel geometries will be formed using soft lithography on the 4′′ silicon wafers after baking at 1000°C for at least 10 hours to get at least 1 *μ*m thickness of an oxides layer. The wafers are vapor coated with hexamethyldisilazane (HMDS) adhesion promoter. After the mask is completed, photoresist (AZ ECI #3012, AZ Electronic Materials, Branchburg, NJ, USA) is poured on the wafer around 2.5 mL and spin coated at 4000 rpm for 30 s (expected thickness less than 0.8 *μ*m layer), and then the wafer is baked at 90°C for 1 minute. After exposure, native oxide is removed with a 20% KOH solution dip at 80°C for 2 hours and 5 hours so that 100 and 250 *μ*m etch depths for the microchannels will be achieved. The final step of the wafer fabrication is to remove the oxide by using a BOE etch. Lastly, the assorted microchannels will be assembled to the PDMS reservoir and sealed using the O_2_ plasma etching processes in accordance with 600 mTorr pressure and 20 W power for 35 s.

## 3. Results and Discussion

**Figure 5:**
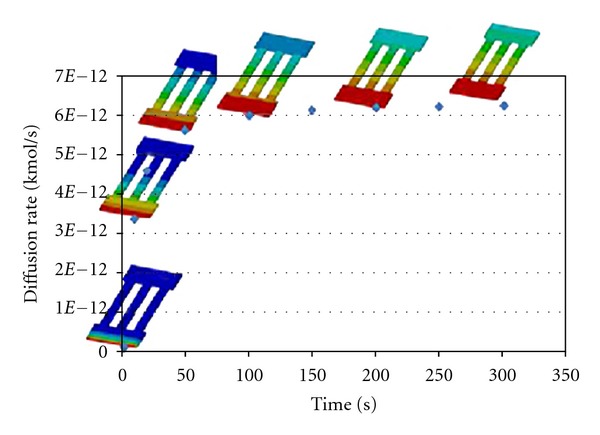
Simulation results of drug diffusion through a straight-type microchannel configuration.

**Figure 6:**
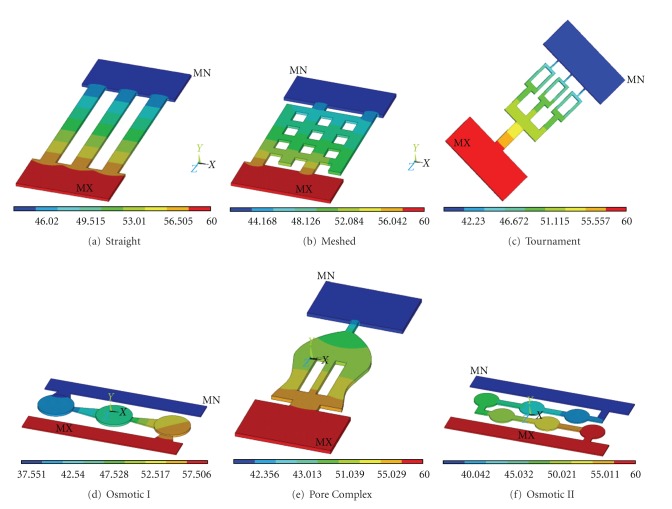
Simulations of drug diffusion at 50 seconds through various microchannel configurations.

**Figure 7:**
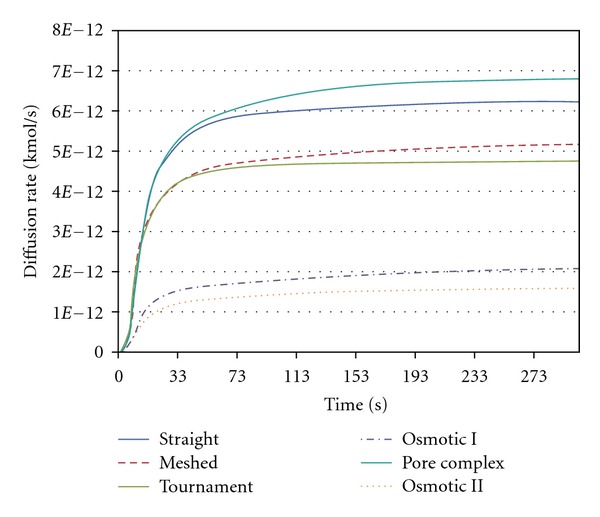
Diffusion rate for various microchannel configurations considered.

Several simulations of drug diffusion rates from various microchannel configurations were carried out. The result of diffusion rate through typical straight microchannels is shown in Figure 5. The length and width of the straight microchannel for this simulation are 8 mm and 500 *μ*m. In this simulation, we assumed that the drug diffuses from the drug reservoir, as we discussed using Fick's Law, which states that molecules will diffuse out to an area of low concentration from an area of high concentration through microchannels. The movement of drug across a micro channels in a manner driven solely by the concentration gradient. Flow field reached at the end of the channel within 38 seconds. Fully developed flow, with sustainable diffusion rates, occurred at approximately 150 seconds. The results of drug diffusion at 50 seconds through various microchannel configurations considered are shown in Figure 6. It is interesting to note that different microchannel configurations will give rise to different diffusion rates. The drug diffusion rate as a function of time for various microchannel configurations is presented in Figure 7. It can be seen from Figure 7 that each of the microchannel configurations exhibits different diffusion characteristics in terms of drug diffusion rates. Initially, there is a drastic increase and after a certain time, the diffusion rate is almost constant. If the drug was to be delivered at a constant rate over a one-hour period then the inlet flux of straight microchannel would be 6.25 × 10^−12^ kmol/s. Over the first second, there is a rapid increase of diffusion rates up to approximately 1.24 × 10^−13^ kmol/s and then a more gradual increase to approximately 6 × 10^−12^ kmol/s after 105 seconds. Overall, each of the microchannel configurations can deliver the drug at different diffusion rates.

**Figure 8:**
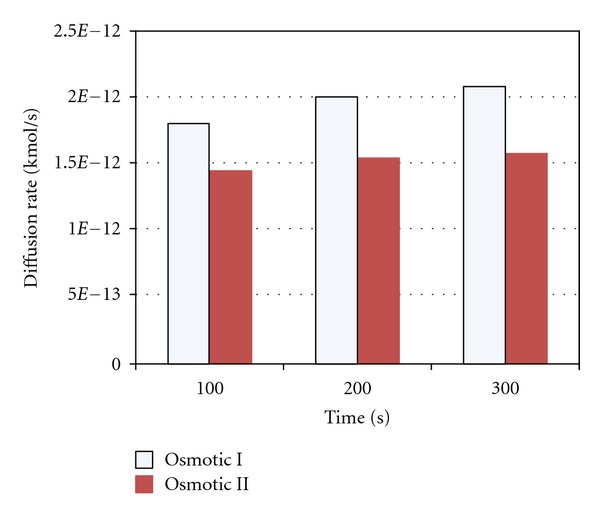
Diffusion rate comparisons at different times through the microchannel designs (osmotic I and osmotic II) for ocular drug delivery applications.

**Figure 9:**
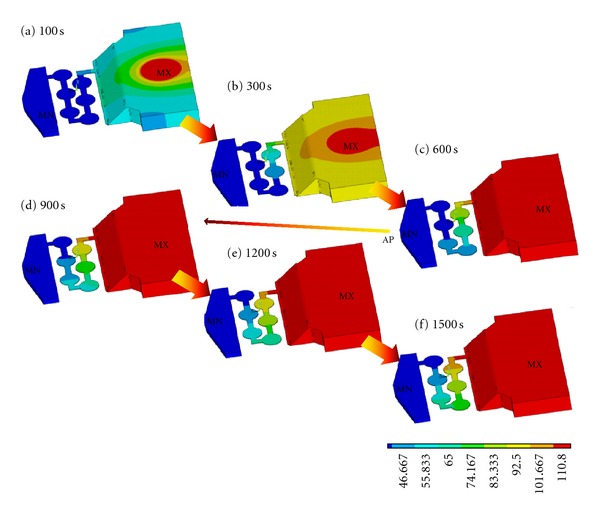
Illustration of drug diffusion through the osmotic II microchannel from the reservoir.

Based on the results obtained through various microchannel configurations, the designs: osmotic I and osmotic II best satisfied the diffusion rate specifications (less than 0.07 nL/min) for the developed ocular drug delivery device. These results are presented in Figure 8. In order to demonstrate the diffusion through the entire device, an analysis is carried out using the osmotic II microchannel and the reservoir for 1500 seconds using ANSYS software. It is assumed that the thermal conductivity and specific heat and density were set as 0.6 W*·*m^−1^ *·*K^−1^, 4181.3 J/kg*·*K, and 850 Kg/m^3^, respectively, for simulating the diffusion through the device. No thermal conduction is considered at the surrounding wall of a device. The temperature of 120°C at the inlet (center top of reservoir) and 37.5°C at the outlet is applied for this simulation. The result of diffusion at various times is shown in Figure 9. It can be seen from Figure 9 that a very slow diffusion occurs most likely at the narrow channel paths. This demonstrates that the developed microdevice is capable of delivering the drug through the osmotic II microchannel configuration. 

**Figure 10:**
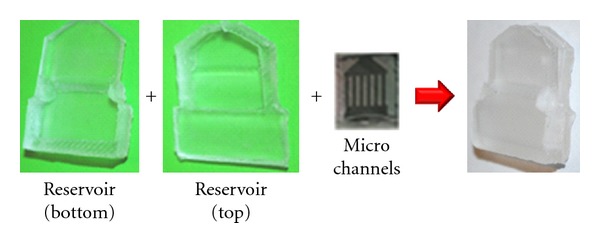
PDMS-fabricated drug delivery device concept.

The results obtained from the simulations confirmed that the microchannels have the potential to be used as a drug delivery system depending on desired flow rates and drug concentrations. The proposed device can produce a constant delivery rate, which is favorable to the treatment of eye disease. Diffusion rates can be customized to obtain effective levels by varying height, width, and length of microchannels. The overall fabricated device is shown in Figure 10. Currently, the functionality of the device is being explored and will be tested in future.

## 4. Conclusions

A microdevice concept for ocular drug delivery is proposed in this paper. The design involves development of an implantable device with micro-/nanochannels with top and bottom covers. Six different channel configurations were developed and analyzed for their diffusion characteristics. Based on the results obtained, channel design of osmotic I and II satisfied the diffusion rates required for ocular drug delivery. In addition to design simulations, the top and bottom covers were fabricated from PDMS through replica-molding techniques. The microchannels along with top and bottom covers were all integrated into the device. Currently, the device is being tested for its functionality and diffusion characteristics. However, there are significant challenges related to achieving reliable and sustainable integration, bonding, diffusion of the drug into channels, and controllability. The test evaluation will be performed measuring the change in pH of a neutral solution using a strong citric acid; it can be diffused out through the device. These challenges are being addressed and will be presented in our future work.

## Figures and Tables

**Table 1:** Diffusion coefficient of drugs for the AMD.

Drug	Drug name (type)	Diffusion coefficient (cm^2^/s)	Average dosage (nL/min)	Injection amount/periods	References
Antiangiogenic	Macugen (pegaptanib sodium)	3 × 10^−7^	0.2083	3 mg/10 days	Swanson [12]
	Lucentis (ranibizumab)	2.08 × 10^−7^	0.0124	0.5 mg/month	Molokhia et al. [13]
	Intravitrea Avastin (bevacizumab)	1.25 × 10^−7^	0.0289	1.25 mg/month	

Synthetic corticosteroid	Fluocinolone	2.3 × 10^−7^	0.0744	15 mg/20 weeks	Li et al. and Jaffe et al. [14, 15]
	Acetonide				
